# Screening Outside the Catalytic Site: Inhibition of Macromolecular Inter-actions Through Structure-Based Virtual Ligand Screening Experiments

**DOI:** 10.2174/1874091X00802010029

**Published:** 2008-03-10

**Authors:** Olivier Sperandio, Maria A Miteva, Kenneth Segers, Gerry A. F Nicolaes, Bruno O Villoutreix

**Affiliations:** 1 Inserm U648, University of Paris 5, 45 rue des Sts Peres, 75006 Paris, France; 2 Department of Biochemistry, Cardiovascular Research Institute Maastricht, Maastricht University, the Netherlands

**Keywords:** Virtual screening, structure-based drug design, drug discovery, protein-protein interaction, protein-membrane interaction

## Abstract

During these last 15 years, drug discovery strategies have essentially focused on identifying small molecules able to inhibit catalytic sites. However, other mechanisms could be targeted. Protein-protein interactions play crucial roles in a number of biological processes, and, as such, their disruption or stabilization is becoming an area of intense activity. Along the same line, inhibition of protein-membrane could be of major importance in several disease indications. Despite the many challenges associated with the development of such classes of interaction modulators, there has been considerable success in the recent years. Importantly, through the existence of protein hot-spots and the presence of druggable pockets at the macromolecular interfaces or in their vicinities, it has been possible to find small molecule effectors using a variety of screening techniques, including combined virtual ligand-in vitro screening strategy. Indeed such in silico-in vitro protocols emerge as the method of choice to facilitate our quest of novel drug-like compounds or of mechanistic probes aiming at facilitating the understanding of molecular reactions involved in the Health and Disease process. In this review, we comment recent successes of combined in silico-in vitro screening methods applied to modulating macromolecular interactions with a special emphasis on protein-membrane interactions.

## INTRODUCTION

Among the over 30,000 unique protein sequences present in the Human proteome, only 1% have been successfully targeted with small drug molecules [1]. Most recent small molecule drug discovery initiatives are been used to develop and/or expand the already existing pre-clinical and clinical pipelines focusing on G-protein coupled receptors, nuclear receptors, ion channels and enzyme active sites [2]. However, key functional regulations of numerous biological processes involved in the pathogenesis of diseases can also be achieved through targeting protein-protein interactions (PPI), or protein-membrane, protein-RNA or protein-DNA interactions. For example, a recent study has estimated that ~29% of proteins that bind another protein are potentially druggable [3]. Maximizing the range of proteins that can be targeted with small-molecule drugs is critical to fully exploit the wide variety of molecular targets that are encoded by the human genome [1]. In fact, increasing the number of potential targets will soon become critical given the fact that there is a significant number of disease indications with unmet clinical needs. The call for alternative drug targets is a problem that is also likely to worsen due to the emergence of clinical complications with current therapeutic drugs, world population aging and the birth of new diseases. These observations lead to two overlapping concepts and needs, (i) identifying and characterizing more targets, new targets or targeting presently known macromolecules at hitherto untargeted sites, and (ii) trying to understand the connections between the target space and the chemical space through, for instance, ongoing chemogenomics/chemical biology projects [4-6]. Along this line of reasoning and toward the identification of mechanistic chemical probes or toward leads that could be optimized into drugs, drugging PPIs and/or exosites instead of catalytic sites via small drug-like molecules can be considered as a paradigm shift in drug discovery. From a biological standpoint and in favor of such initiatives, the concept of inhibiting PPIs has been validated through the use of, for example, mini-proteins, antibodies or antagonist peptides (and peptide aptamers), some of which have become highly successful drugs [1]. However, in most situations, it would be desirable to design “drug-like” inhibitors instead of therapeutic proteins or peptides. These latter therapeutic molecules usually have to be infused, a situation that is not comfortable for patients nor clinicians while the costs associated with such therapies is already intractable in many countries. Not in favor of the ideas of targeting macromolecular complex formations or exosites are claims that impeding protein-protein associations is difficult for energetic reasons or that the small organic compounds able to perform such action will fall outside the range of the so-called “drug-like” profile and therefore could not be administrated orally [2, 7]. Even if these observations are partially correct, among the 40,000 to 250,000 known or predicted PPIs in humans [8, 9], there are strong evidences that it should be possible to target, with drug-like compounds, a sizable number of these interfaces [10]. In fact, several observations provide hope for finding small molecules that target protein-protein interfaces. Although the interfaces are large, mutational studies show that only a small subset of residues contributes significantly to the free energy of binding [11]. Moreover, peptides selected to bind to one protein partner by phage display or through computer aided rational design approaches often compete with the other protein partner. As such, tight binding at protein-protein interfaces does not necessarily require extended surface contacts [11].

PPI networks embody a system-level description of the proteome and will be invaluable for systems biology approaches to model complex processes such as signaling pathways [9]. Knowledge about protein-protein interactions will enable a better understanding of the physiology of signal transduction at the molecular level and the manifestation of multi-factorial diseases that originate from disturbances of overlapping pathways. A recent evaluation of the number of PPI in the interactome gives 250,000 interactions with only 5-10% of pair-wise interactions being already charted [9]. Of these described interactions, one cannot indicate how many have a real and direct therapeutic potential. However, with tangible success stories such as p53-MDM2, Ras-Raf, XIAP-smac (and several others), we foresee that these may be the forecast of more small molecule-PPI modulators to appear in the near future.

While antagonizing macromolecular interaction with chemical compounds is recognized by many to be challenging but possible, some researchers are still critical as to the feasibility of such projects and as such the question/concept is still under debate. The principal argument concerns the size of the small compounds with respect to their natural competitors (e.g., 1000 times bigger) and the incapacity of small molecule to disrupt protein-protein complexes once they are formed. However, if one considers that binding in general is as an equilibrium process between isolated monomers and a multimeric complex, it is no longer reasonable to think that a small molecule can not bind specifically to one partner and prevent/stabilize association [12]. Yet, clearly, different types of protein complexes have been observed, homo- and hetero-complexes (i.e., the interaction occur between identical or non-identical chains) and obligate and non-obligate complexes (a related view refers to interactions as transient or permanent). Well-known examples can be, for a permanent complex, the interleukin 8 homodimer (PDB code 1il8), and for a non-obligate complex, the cytochrome c peroxidase associated with cytochrome c (PDB code 2pcb). Also, depending on the type of complexes (permanent, transient…), the nature of the interface usually differs, from somewhat hydrophobic/aromatic to a mixture of hydrophobic/aromatic and polar properties. As such, it is possible that small molecules could only target efficiently some types of complexes, but recent studies do not support this view and further work is required to fully address this question [10].

Protein-protein interactions represent a vast and diverse group of targets for therapeutic innovation [11], yet other kinds of macromolecular interactions are likewise abundant in living organisms and many of them have been neglected over the years. In this article, we will comment on the discovery of drug-like molecules inhibiting the transient interactions between a protein and an appropriate membrane surface. However, while it is important to screen as many targets as possible, it is also important to keep in mind that drug discovery is a costly and time-consuming process [13] and that screening thousands of targets and millions of small drug-like molecules may not be feasible through conventional approaches such as High Throughput Screening (HTS). Consequently, methods that reduce the costs and speed-up the overall process have to be applied, and among those*, in silico* methodologies are becoming well-established in the field of drug discovery and have been applied successfully to numerous targets [14-18].

Here, we will briefly introduce the concept of targeting regions located outside the catalytic sites and illustrate this point through analysis of recent developments in the protein-protein interaction field. The opportunities that are created in terms of new areas of therapeutic innovation or better understanding of molecular events are discussed. Then, we will focus on transient protein-membrane interaction; a new class of targets that we think should be investigated as an alternative route for the design of novel therapeutic agents. We will take as example our recent proof of concept study, carried out on the non-enzymatic coagulation factor V [19]. Along the present review, we will also comment on the roles that *in silico* tools can play to help prioritize targets and small molecules, thereby facilitating the drug discovery process and/or chemical biology projects.

## Screening Regions Outside the Comfort Zone in a Cost Effective Fashion

Conventionally and during the last 15 years, the search for lead compounds has involved HTS screening of all possible chemicals available in compound collections. Although the method is attractive, the hit rates are generally disappointing considering the costs, the time and the need of large quantities of biological materials (e.g., purified proteins, small compounds…) [20]. The development of virtual screening methods allow for a more rational and efficient screening in many situations and indeed, virtual screening tools are more and more applied prior to HTS experiments. Yet, all scientists working in the drug discovery field know that in order to succeed, a combination of methods is usually necessary and that drug discovery requires multi-disciplinary team-work.

**Fig. (1):**
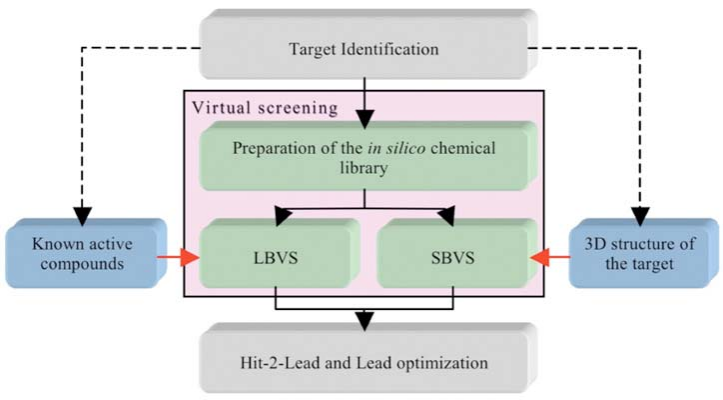
The two components of virtual screening. The selection of LBVS and/or SBVS is based on the amount and type of information vailable on the target at the beginning of a screening campaign.

While *in silico* screening strategies still suffer from obvious limitations, many new hits have been identified after application of these computer tools.* In silico* techniques usually involve the screening of chemical compound libraries (i.e., in general the compounds are available or can be purchased, although in some cases the compounds can be virtual and will thus have to be synthesized should they be selected by the process). These techniques are used to predict, instead of measuring, the potency of a small molecule on a given bio-molecular target. Depending on the information available at the beginning of a screening campaign (e.g., crystal structure of the target, and/or knowledge of previously determined chemical compounds acting on the desired target) two strategies can be applied: structure-based virtual screening or SBVS (i.e., docking/scoring) [14, 21-23] or ligand-based virtual screening or LBVS [24-35] (Fig. **1**). The first steps of SBVS approaches involve docking computations. These consist of placing the small molecules that are present in the (virtual) chemical library into a (known or predicted) binding pocket such that the predictions of a likely pose and of a relative affinity can be established at a later stage. LBVS, on the other hand, make use of previously identified chemical compounds to identify new ligands based on 2D and/or 3D similarity searches, and in this case, the 3D structure of the target is not required. In some projects, it can be rewarding to combine both SBVS and LBVS with other methods, such as NMR (Nuclear Magnetic Resonance), crystallography and site directed mutagenesis. The projects and the first results obtained after initial screening experiments usually guide the selection of an appropriate set of methods to be used.

**Fig. (2):**
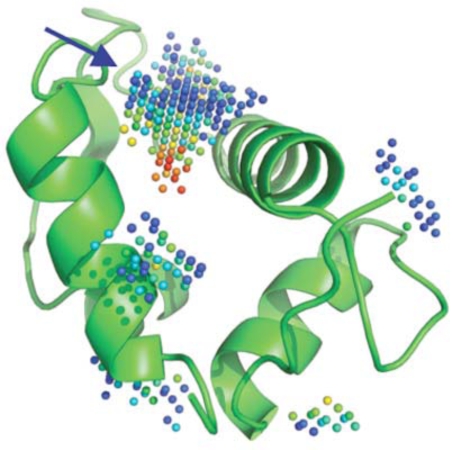
Five cavities detected by PocketPicker [37] on MDM2. The predicted deepest and largest pocket (blue arrow) is indeed the key binding site for p53 and small molecules impeding p53 binding. Red spheres represent regions that are more buried in the protein interior as opposed to blue spheres, that become fully solvent exposed when the color shift to navy blue

Overall, *in silico* and HTS methods have been very successful in screening catalytic sites, in part because the pocket to be screened is usually “druggable” (e.g., well formed binding region with a topology and physico-chemical properties compatible with the tight binding of a small molecule), although even if druggable, there are usually no easy targets. The situation can be significantly different though when screening protein-protein interfaces. For PPI, and in contrast to conventional druggable targets, the small molecule inhibitors or stabilizers can be thought, at first, as having to interact or interfere with a large interface area, often lacking a well formed cavity or cleft [1]. Because of this preconceived idea, the rational design of small molecules able to perturb PPI has long being thought of as an unreachable goal. However, today, several successful examples have been reported [36] confirming the hypothesis that small non-peptide molecules can interfere with PPIs. Indeed, even at protein-protein interfaces, druggable pockets displaying characteristics closely related to the ones forming catalytic sites can be found (Fig. **2**).

**Fig. (3):**
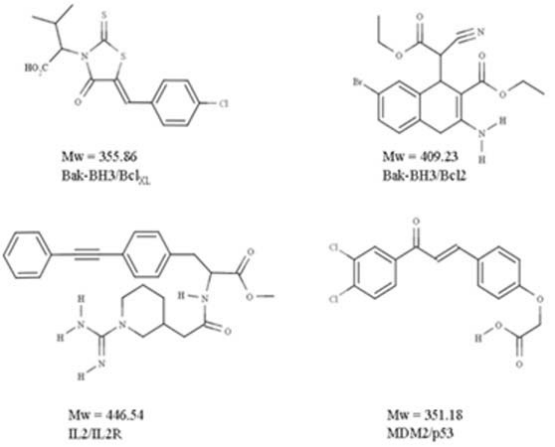
Examples of “drug-like” protein-protein antagonists.

Crude structural analysis of experimental protein-protein complex could initially give the impression that the topological features present within the interface will not favor the efficient and selective binding of a small molecule able to interfere with the formation or the stabilization of a macromolecular complex. Clearly, the interaction surface involved is rather large (700-1500 Å^2^) and the binding surfaces tend to be relatively flat [36, 38]. However it was suggested that the size of the interaction patch of residues present at the interface is not systematically meant to be covered by the small molecule [39], but rather that the binding of a drug in one specific region of the interface could be enough to perturb complex formation or to the opposite, stabilize an interaction. For example, the subset of the interface that contributes to high-affinity is much smaller [39] than the interface itself and it is not significantly bigger than that of a standard “drug-like” molecule [40-42] (Fig. **3**). Consistent with this view, the existence of “hot-spots” have been introduced by Wells and co-workers [40] some 15 years ago. Using alanine scanning mutagenesis experiments on human growth hormone and its receptor, they demonstrated that even though the binding interface is quite large, only a subset of the residues involved is directly implicated in the bulk of the binding energy. Therefore, the perspective of finding small molecules that specifically interact with those hot-spots revived the pursuit of protein-protein interaction antagonists (and possibly agonists). Since, a lot of work has been put through the characterization of the hot-spot regions.

For instance, Bogan and Thorn have examined 2325 alanine mutants for which changes in the binding free energy have been measured, and showed that the energetic contributions of the individual side chains did not correlate with their buried surface [43]. In several cases, a set of energetically unimportant contacts surrounded the hot-spots and appeared to occlude bulk-solvent access by formation of what was called an “O-ring” [44]. From their work it was furthermore concluded that certain amino-acids were more present in the observed hot-spots, noticeably tryptophan (21%), arginine (13%), and tyrosine (12%) than other amino acids such as leucine, methionine, serine, threonine, and valine each of which accounted for less than 3% of all hot-spot residues [45]. However, these observations do not apply to all complexes, it for instance depends if they are permanent or transient. Stites *et al *[46]**studied the thermodynamics of protein-protein association and pointed out that hydrophobic interactions generally provide the key driving forces for many protein-protein complex formations, suggesting that it could be difficult to counteract this event with a small drug-like molecule. To bind, a small compound usually requires a cavity, that pre-exists, at the interface, or that is formed during the protein-drug or protein-protein interaction. The presence of a cavity at, or next to the interface, containing (or in the vicinity of) the hot-spot residues is thus usually required to launch *in silico* screening experiments. Other difficulties with targeting PPIs come from possible flexibility at the interface. While active site regions tend to be relatively rigid and well formed, protein interface regions can undergo conformational changes that are rather difficult to predict, thereby, in some situations, impeding the rational design of drug-like inhibitors or stabilizers through available *in silico* procedures. Thus, two main problems generally have to be solved by the drug designers aiming at interfering with PPIs. First, the identification of a suitable region on the 3D structure of a protein or of a complex and second a way to investigate the flexibility of the region (this can also apply to catalytic sites, of course but it may be less acute). Interesting zones can be pinpointed through hot-spot/binding site predictions, data mining, structural analysis, protein docking combined with for instance site directed mutagenesis experiments [47-49]. Next, the ability of a protein to bind another protein usually involves a series of pharmaco-topological complementarities that can require minor to important conformational changes [50, 51]. These changes can be very difficult to assess experimentally or *in silico*. Nevertheless computer methods such as normal modes, gaussian network model, and molecular dynamics can be applied [52].

## Protein-Protein Inhibition Success Stories

In spite of the difficulties mentioned above, several previously reported small molecules are able to interfere with PPIs. These encouraging studies are likely to be followed by many such investigations in the near future, paving the way for the design of new therapeutic compounds. The chemical compounds that can mediate the perturbation of the interaction act either directly, through an inhibition of the protein-protein binding or indirectly, via the binding to an allosteric site and the distant induction of a perturbation signal that will promote conformational changes and impede binding. In oncology, a recent set of exiting targets has emerged with reported inhibitors showing sufficient potency and intracellular activity to address the biological challenge and to start acting on the diseases. Most of the recently identified targets in oncology are characterized by the interaction between a linear region of one partner and a hydrophobic cleft on the other [7]. Several of the biological systems concerned by these investigations have focused on cancer, but not exclusively [53]. Specific examples can be cited here, like MDM2-p53, Bcl-XL-Bak, IL-2-IL-2Rα, LFA-1-ICAM1, Ras-Raf, XIAP-smac, TNF-TNFRc1 [38].

## Applications of in Silico Screening to PPIs

In the following, we will limit ourselves to a situation where structure-based ligand screening methods can be applied. In fact HTS is not always well-suited for screening protein-protein interaction because of difficulties setting-up the assays, among others. As such, a better solution in terms of costs and efficacy could be to first use *in silico* screening methods followed by low or medium throughput assay formats.

A well-known example of a macromolecular complex that has been investigated through computational studies is the p53-MDM2 complex. In this case, the flexibility-induced-fit problem is not really present and the druggable cavity appears to be pre-formed on one partner, allowing the direct use of virtual screening strategies. The p53 tumor suppressor is central to the regulation of cell cycle progression, DNA repair, and apoptosis and is an attractive therapeutic target because its tumor suppressor activity can be employed to eradicate tumor cells [54]. The complex is characterized by the interaction of a short helix in the N-terminus of p53 and a small, deep hydrophobic pocket in MDM2 [55]. Typically, the pocket in MDM2 represents an attractive site to block the p53-MDM2 interaction. Lu *et al *[56]**have indeed used this pocket and available crystal structures with cocrystalized small-molecules to perform a two-step computational study. First, they derived from the cocrystalized small-molecules a three-point-pharmacophore based on three hydrophobic spots, that they used to screen the National Cancer Institute (NCI)’s 3D database in order to obtain an initial list of hits. Second, they docked these hits to the p53 site in MDM2. The study has allowed the identification of a novel inhibitor with a K_i_ of 120 nM with respect to a K_i_ of 6670 nM for the natural binder p53 in the same assay.

However, it should be born in mind that some other protein complexes are much more challenging to target. A clear example that illustrates the difficulties of targeting PPIs (but which also applies to catalytic pocket to some extent) is the case of the IL-2-IL-2Rα receptor complex. In this macromolecular complex, the presence of an equilibrium between several conformations has been observed and small molecules have been determined that prevent the interaction between IL-2 and its receptor IL-2Rα by binding to IL-2. Interestingly, the inhibitory efficiency of the ligands is due to binding to small cavities on IL-2 inaccessible to IL-2Rα. The ligands possess a similar but more localized electrostatic field as compared to the IL-2Rα receptor, and target the same hot-spots than the one used by the receptor for binding IL-2 [57]. IL-2Rα completely envelops the footprint covered by these small-molecule competitive inhibitors. However, the conformation of these hot-spots on the IL-2 side is different depending on which partner binds to IL-2. Therefore, there are multiple tight binding complex conformations at these common and adaptive hot-spots [57]. This result allows researchers to envision more drug discovery perspectives than the availability of static structures only permits, however a rational and cost-effective way to perform such tasks remain challenging. The kind of perspective we can draw from the IL-2-IL2Rα case is two-fold. First, the induced-fit of biomolecules when bound to a given partner can be specific for this partner, which brings more complexity to the system that is being studied. Second, the X-ray structure may in case of induced-fit not be the ideal source of topological information in terms of what is to be called a binding site *per se* (i.e. a cavity large enough to fit a small molecule). This means, that the sole rigid surface of the receptor crystallographic structure cannot be the ultimate element to determine whether a structure-based project is feasible or not, but rather it could be wise to combine the information with previously mentioned computer tools, such as normal mode analysis and molecular dynamics. In fact, in order to try to address the flexibility problem, the main protocols currently applied can be summarized as follow [14]. The first approach involves the use of soft potentials, where some atom overlaps are tolerated. Another way is to dock on multiple structures (experimental or simulated) of the receptor. The ultimate approach would be to explicitly consider protein and ligand flexibility during docking. Yet, this latter approach is very computationally demanding and, for the time being, basically no packages are able to address the problem efficiently in the context of high-throughput docking, while molecular dynamics could be run on each docked ligand and somewhat could allow both, flexibility of the receptor and of the ligands. The problem of performing docking against multiple receptor conformations lies with how to deal with the ranked lists since for each structure of the receptor, the docking/scoring procedure can propose over 1000 likely molecules. If this computation is performed on 3 structures, one may have a list of 3000 molecules to test experimentally with possibly very few consensus compounds. The merge-and-shrink procedure has been recently proposed to address this problem [58]. With this approach, for each compound, the best rank -not the best score- is kept. This was shown efficient on some targets but, in general, to use this approach, one needs known actives to test the protocol and calibrate the computations. The merge-and-shrink protocol can thus be efficient in some cases or dilute the correct results for some targets. As such, much work is needed to address this difficult question of flexibility from the receptor side. Interestingly, a recent molecular dynamics study investigating transient pockets on protein surfaces directly located at the PPIs has been reported [38]. The authors investigated BCL-Xl, IL-2 and MDM2 and proposed a new pocket detection protocol. They observed that the native inhibitor-binding pocket was partly present in the unbound proteins. They performed 10 ns molecular dynamics simulations and scanned, on many structural snapshots collected along the trajectory, the surface of the proteins, in search of transient or more permanent cavities. They noticed that some cavities were opening up within 2.5 ps and that most of them appeared multiple times. In the druggable region, they noticed that pockets of similar size as compared with the experimental structures in complex with the ligand could be generated. Docking was successfully performed into these transient pockets, suggesting that this protocol is viable to study protein interfaces with the goal of designing antagonists and possibly stabilizers although it remains to be fully demonstrated if this approach is viable in a real life situation, with a large compound collection and the need to select a small list for in vitro testing. Thus, it would be interesting to apply these new methods on the IL-2-IL-2Rα receptor complex in the context of high-throughput docking/scoring in order to see if it is possible to find interaction inhibitors *via* such protocols.

## Rational Inhibition of Transient Protein-Membrane Interactions

Based on recent investigation of PPIs and their potential as targets for therapeutic interventions, one might consider targeting other kinds of macromolecular interactions such as transient protein-membrane interaction. In our recent study of the interaction between the discoidin C2 domain of coagulation factor Va and the platelet membrane [19], we suggested to enlarge the vision we have regarding the development of therapeutic compounds by applying the initial steps of the drug discovery machinery to this hitherto neglected interaction class.

**Fig. (4):**
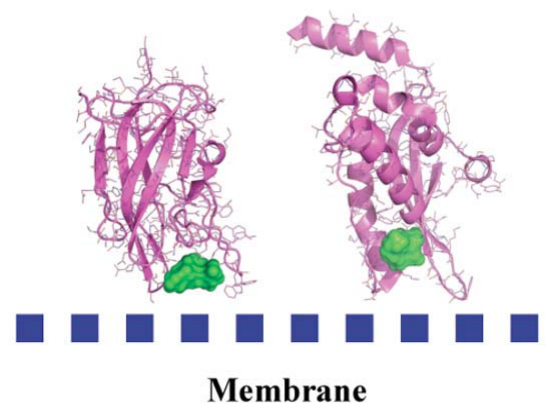
Detected cavities (green) with the computer tool PASS [66], on Factor Va (left) and the PX-domain (right).

Research in the past decade has revealed that many cytosolic proteins are recruited to different cellular membranes to form protein-protein and lipid-protein interactions during cell signaling and membrane trafficking. Membrane assembly of these peripheral proteins is mediated by a growing number of modular membrane targeting domains, including C1, C2, PH, FYVE, PX, ENTH, ANTH, BAR, FERM, and tubby domains, that recognize specific lipid molecules in the membranes [59]. However, the mechanisms by which these domains and their host proteins are recruited to and interact with various cell membranes are only beginning to be unraveled [59]. Among these protein domains, we analyzed the structure of several protein domains known to be involved in membrane interaction, namely, the discoidin C2 domain of coagulation factor Va [60], plasma beta-2 glycoprotein 1 [61], cyclooxygenase [62], PX domain [63], and the tubby-protein [64]. We found, in agreement with a recent bioinformatics study [65] that these membrane-binding regions possess specific chemical and topological properties such as cationic patches that are surrounded by aromatic and aliphatic clusters capable of interaction with phospholipids head groups. In addition, we noticed that a druggable pocket [19] (Fig. **4**) was present at the expected protein-membrane interface region (or nearby) which suggests that inhibition of membrane-binding (possibly stabilization but this needs new investigations) could be addressed via combined *in silico-in vitro* procedures.

Interestingly, the size of the predicted binding pocket envelopes for these five membrane-binding proteins within the membrane-binding region ranges from 200 to 600 Å^3^, values commonly computed for proteins co-crystallized with drug-like ligands [67].

**Fig. (5):**
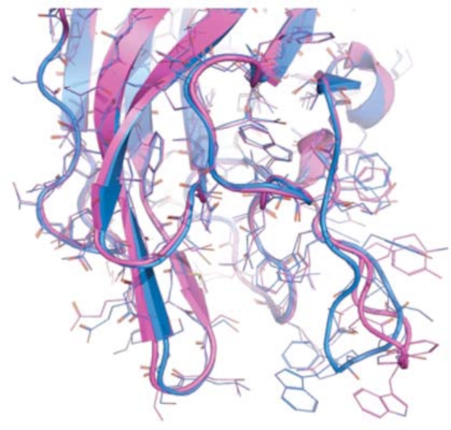
The two known conformations of the discoidin C2 domain of coagulation factor Va, open (magenta) and closed (blue).

As a proof of concept, we decided to study the discoidin C2 domain of FVa, and tried to identify drug-like molecules that have the capacity to prevent the interaction of this domain with a relevant membrane surface (Fig. **4**) using *in silico* screening, functional assays and surface plasmon resonance (SPR) [19]. The C2 domain of coagulation FV can be considered as a representative protein domain involved in calcium-independent membrane binding. This domain is closely related, structurally and functionally, to the C2 domain of factor VIII (FVIII, a protein involved in hemophilia A) [68]. Although this domain is named C2, this region of FV or FVIII is structurally unrelated to the so-called C2 domain of for instance cytosolic phospholipase A2. FV and FVIII are homologous molecules that share a domain architecture A1-A2-B-A3-C1-C2. The binding of coagulation FV (via at least its C2 domain) to activated membranes of circulating platelets is essential for the expression of its functions in coagulation. Upon activation by thrombin, activated FV (FVa) acts as a cofactor of activated factor X (FXa) in the prothrombinase (PTase) complex, which converts prothrombin to thrombin on an appropriate phospholipid surface [69]. Excess thrombin formation can lead to thrombotic events [70], suggesting that small inhibitors of FV-membrane interactions could be the starting point for the development of a novel class of antithrombotic drugs. The FV C2 domain comprises a distorted jelly-roll β-barrel motif consisting of eight major anti-parallel strands arranged in two β-sheets. Several loops, presenting hydrophobic/aromatic residues, facilitate immersion of the C2 domain into the membrane [71]. Specific interactions with phosphatidylserine (PS) head groups have been postulated to occur in the groove enclosed by these membrane-binding loops [60]. This zone is surrounded by several basic residues, which facilitate the formation of an encounter complex with the negatively-charged membrane phosphate groups via electrostatic steering, and that anchor the module into the membrane bilayer. The FV membrane-binding loops can assume an open or a closed conformation (Fig. **5**) and it is believed (but not proven) that the closed form has low membrane affinity whereas the open form is suitable for membrane interaction. In the open crystal form, the exposed indoles of Trp 2063 and Trp 2064 contribute to the immersion of the module by interaction with the apolar membrane core. The nearby-predicted druggable pocket is lined with polar/basic/aliphatic/aromatic side chains (Ser 2183, Gln 2085, Lys 2060, Trp 2068, Met 2120, Ser 2115, Leu 2116, Arg 2080) ideally arranged for interactions with lipid headgroups.

**Fig. (6):**
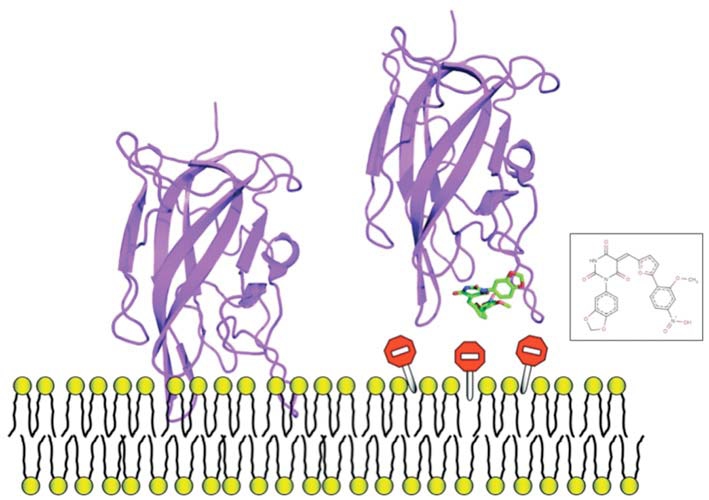
Example of one small molecule identified in the FV-membrane study that impedes protein-membrane interaction, it can be used as a mechanistic probe to investigate this molecular event or, could be used as a starting point to develop anticoagulant molecules.

SB-VLS methods help to prioritize large compound collections prior to experimental testing. We applied our validated multi-step SB-VLS protocol [72] to both, the open and closed C2 crystal forms and selected the best 500 molecules for each receptor form (i.e., we decided to screen around 1000 compounds in total). This hierarchical screening protocol starts with using a relatively large compound collection (of about 500,000 molecules in 2D) obtained from a chemical vendor (in the present case it was ChemBridge [73]). Some molecules that do not appear to have a drug-like profile or that contain reactive groups are removed using several soft ADME/Tox filtering steps [74]. It is important to note that at this level of investigation, the filtering was relatively soft in order to keep a significant number of molecules for the subsequent phases of the procedure. After this ADME/Tox step, about 200,000 molecules were removed and the remaining compounds were transformed to a multiconformer collection in 3D. Each compound was docked as a rigid body and the molecules that had the best shape complementarity with the receptor were kept for an additional flexible docking step. This latter used an incremental construction algorithm as implemented in the Surflex package [75, 76]. Subsequently, the highest-ranking molecules in term of predicted affinity (scores) were selected for experimental assays. We screened experimentally 1018 molecules and we initially studied their ability to inhibit prothrombin activation. Nine molecules were identified that inhibited a prothrombinase assay by more than 99% at a concentration of 100 μM. Two molecules were inhibiting the assay system via a membrane-independent mechanism but seven molecules remained, apparently selectively inhibiting the FV-membrane interaction (see an example Fig. **6**). To confirm that these compounds were impeding the association between of FV C2 domain with the membrane, we performed surface plasmon resonance (SPR) experiments. Results of these direct binding analyses confirmed that the small molecules were indeed inhibiting the protein-membrane interaction and we noticed that four out of the seven hit compounds also inhibited membrane binding of the structurally related blood coagulation FVIII.

It is interesting to note that the only protein-membrane HTS study published to date was performed on factor VIII, with a compound collection subset of 10,000 molecules extracted from the ChemBridge library [73]. Thus, after completion of our FV study, we decided to perform control *in silico* experiments on the FVIII C2 domain to further validate the concept of using *in silico* screening computations before in vitro experiments when a limited budget is at hand. We retrieved the X-ray structure of the FVIII C2 domain and applied the same VLS protocol as was used for FV, including the FVIII inhibitors of ref. [77] in our 300,000 ChemBridge ADME/tox filtered compound collection. Since the FVIII loops important for membrane binding are in an open conformation, we performed rigid body docking and flexible docking on this X-ray structure. We identified 5 compounds out of the 10 found by HTS in the top 1000 Surflex list. In addition, with the C2 domain of FVIII as a target we also found 3 molecules that had previously been identified in our FV study and that were shown to cross-react with FVIII membrane binding. Thus we obtained, with our *in silico* approach, an overall hit rate for FVIII of at least 0.8. These results indicate that for FVIII, our strategy is more efficient than the HTS-only approach, since in this case, the hit rate after the initial HTS work was (10 actives/10,000) x 100 = 0.1%.

The advantages of using hierarchical SB-VLS protocols with rigid body docking prior to flexible ligand docking is that the computations run significantly faster (thus can be carried out on only 1 workstation in about 2 weeks) as compared to a full flexible ligand docking of an entire compound collection (about 8 weeks on the same single workstation) while performing better (less noise) or equally well. Possibly, some active molecules are lost because of the hierarchical procedure but from our investigations, real actives are also lost when performing full flexible docking due to approximations in the scoring functions and errors with pose predictions. It would seem indeed that a rigid-body docking step somewhat reduces these artifacts and improves enrichment. The hierarchical approach is also very interesting when several 3D structures of a receptor are available, since again, the computations can be performed on a few workstations in parallel as compared to as using full flexible docking.

## CONCLUSION

Screening of catalytic sites via HTS and *in silico* methods has been performed during 15 years and it is now time to also apply these tools to other target classes, such as protein-protein interactions or protein-membrane interactions. The goal can be to find inhibitors or stabilizers of interactions, either to understand better a molecular mechanism (like in the new emerging chemogenomics projects) or with the intention of developing new drugs. Experimental screening of difficult targets is time consuming and cost intensive but it is known that *in silico* approaches can facilitate the process. In this review, we advocate the use of *in silico* tools in order to target unconventional macromolecules, with a special emphasis on protein-membrane interaction. Therapeutic targets that bind transiently to membrane or protein-protein complexes have been neglected thus far and remain among the most difficult challenges in contemporary drug discovery. In the case of protein-protein complexes, it can be difficult to apply *in silico* methods due to flexibility problems but new protocols have been reported that should facilitate undertaking such projects. In the case of protein-membrane interaction, the situation is also complex. For instance, the mechanism of transient protein-membrane interaction is not fully understood, success stories are extremely rare and generic approaches are missing. Protein-membrane interactions are however crucial in many biological processes as they localize and concentrate key molecular factors on specific cell surfaces. We have shown that combining SB-VLS experiments with *in vitro* assays allowed for the rational discovery of hit compounds that are able to disrupt FV membrane-binding activity in a timely and cost-effective fashion. These molecules are promising leads for the development of *in vitro* tools for hemostasis research and for the design of a novel class of anticoagulant drugs. It is remarkable that small molecules can impede membrane-binding since the binding interface between the membrane-binding domain and the phospholipids is relatively large with, in general, immersion of the proteins several angstroms deep into the membrane bilayers [71]. However, just like in the case of protein-protein interaction [40], the bulk of the binding energy appears to be derived from contacts with just a small number of amino acid residues. We conclude that hierarchical virtual screening approaches in combination with SPR technology are an efficient and generally applicable approach for routinely identifying membrane-binding inhibitors. This strategy could therefore be applied to other relevant membrane-binding proteins in search of the next generation of therapeutics.
